# Research Progress in Carbon Nanotube-Based Cold Cathode Electron Guns

**DOI:** 10.3390/nano15181403

**Published:** 2025-09-12

**Authors:** Jiupeng Li, Yu Tu, Dewei Ma, Yun Yang

**Affiliations:** 1 School of Electronics and Information Engineering, Henan Polytechnic Institute, Nanyang 473000, China; 2000001@hnpi.edu.cn (Y.T.); 2007049@hnpi.edu.cn (Y.Y.); 2 School of Physics, Zhejiang University of Technology, Hangzhou 310014, China

**Keywords:** carbon nanotube, field emission, cold cathode, electron gun, vacuum electron device

## Abstract

Field emission (FE) cold-cathodes have some important characteristics, including instant turn-on, room temperature operation, miniaturization, low power consumption, and nonlinearity. As emitters, Carbon nanotubes (CNTs) exhibit a high field enhancement factor, low turn-on voltage, high current density, high thermal conductivity, and temporal stability. These properties make them highly suitable for applications in FE cold-cathodes. In addition, Carbon nanotube (CNT) cold cathodes have specialized applications in electron beams, which are modulated by high-frequency electric fields and exhibit low energy dispersion. There have been substantial studies on CNT-based cold cathode electron guns with diverse structural configurations. These studies have laid the foundation for the applications of microwave vacuum electron devices, X-ray equipments, flat-panel displays, and scanning electron microscopes. The review primarily introduces cold cathode electron guns based on CNT emitters with diverse morphologies, including disordered CNTs, aligned CNTs, CNT paste, and other CNTs with special surface morphologies. Additionally, the research results of microwave electron guns based on CNT cathodes are also mentioned. Finally, the problems that need to be resolved in the practical applications of CNT cold cathode electron guns are summarized, and some suggestions for future development are provided.

## 1. Introduction

A cold cathode electron gun is a key component used in microwave vacuum electron device sources [1,2], X-ray sources [3,4,5], field emission displays [6,7], scanning electron microscopes (SEMs) [8], electron beam lithographies [9], and other applications [10,11]. In general, a cold cathode electron gun consists of a cathode, grid, focusing electrode, and anode, with the cathode being an indispensable component. Electron guns can also adopt both diode and triode configurations [12,13]. The electron gun can emit a high-quality electron beam with a certain size, trajectory shape, and electron energy to meet the application requirements. The electron beam power can be directly harnessed or converted into radio frequency output power through the electromagnetic interaction structure. Conventional thermionic cathode electron guns need operation at high temperatures, resulting in long start-up time due to heat inertia, high requirements for the cathode structure, and high power consumption, which may limit their further development [14,15,16,17]. In contrast to thermionic cathodes, cold cathodes generate electrons through field emission. Field emission differs fundamentally from thermionic emission in terms of physical principles; it requires no additional energy supply to the emitter. Instead, it employs an intense local electric field to suppress the height and narrow the width of the potential barrier on the emitter surface, which enables electrons within the emitter to escape via quantum tunneling, i.e., electrons are extracted from the emitter. The process is described by the Fowler-Nordheim (F-N) theory [18,19]. FE cold-cathodes exhibit several key advantages, including fast response, room temperature operation, miniaturization, low power consumption, and nonlinearity. These advantages can overcome some limitations of the applications of the thermionic cathodes [20,21,22,23].

Since Iijima reported CNTs in 1991, the FE characteristics and applications of CNTs have aroused great attention [24,25,26]. There are several important reasons why the CNT is suitable for FE cold-cathode materials. Firstly, the CNT emitter with a high aspect ratio has a high field enhancement factor, which is beneficial for electric field enhancement [6]. Secondly, the current density of the CNT emitter is very high. The maximum current density measured for a CNT can maintain 10^7^~10^13^ A/cm^2^ [26,27]. Such a high current density can reduce the electron emission size of the cathode by using a miniaturized FE electron source, ultimately reducing the overall package size of the device, which also provides a way for device assembly on-chips [28]. Thirdly, CNT emitters have the advantages of low turn-on voltage and low power consumption. Furthermore, they also possess high thermal stability, low energy spread, and low-cost synthesis [29,30,31,32]. CNTs have exceptional properties attributed to the C–C covalent bond, one-dimensional carbon nanostructures, and seamless hexagonal network architecture [33,34,35,36]. However, there are also some unfavorable practical factors to consider, such as large emission current, electron emission uniformity of large-area and long-term working stability, when using the CNTs as FE cathodes, which may affect the actual characteristics of CNT cathodes [37,38,39]. In order to fabricate good electron emitters as an electron source, the preparation techniques and voltage driving mode of carbon nanotube cathode have long been developed [40,41,42,43]. For example, CNTs paste emitters were fabricated using graphite nanopowder filler, which raised emission current after sandpaper treatment and showed excellent long-term emission stability [44]. By optimizing the thickness of the LiF/Al coating film on the CNT cathode surface, the FE characteristics of carbon nanotubes can be improved [45]. A double-layer carbon nanotube cathode structure, which comprises a primary cathode and a second cathode, was proposed to increase the effective emission area, the emission current, and enhance the emission stability [46]. The shapes of the CNT emitters greatly affect electron beam trajectories [47].

The quality of the electrical contact between the CNT emitter and the substrate or electrode critically influences the current path and introduces contact resistance. The resistance can significantly affect FE uniformity, high-current performance, and lifetime. The resistance can be controlled by adjusting the thicknesses of the silver, nickel, and tin metal layers on the substrate, as well as through heat treatment [48]. And the heat treatment process can also enhance adhesion between the CNTs and the electrodes to improve FE performance. The aging process can indicate the adhesion of the CNT emitter on the electrode. Moreover, engineering the CNT-substrate interface and using highly conductive substrates can help reduce contact resistance. Different surface treatments on carbon nanotube emitters can effectively modify the surface morphology of CNT emitters to improve the emission property [49]. Additionally, the field screening effect and edge effect are also two important reasons that can greatly influence the emission characteristics of the CNT cold cathode [50,51,52]. Optimization of the alignment and density of CNT emitter arrays by varying tip density, pitch, and spacing can improve electron emission characteristics. The spacing among uniform CNTs should be approximately twice their height, which contributes to the minimization of the screening effect [53]. Jung Sang Suh et al. studied the field screening effect caused by the proximity of neighboring carbon nanotubes (CNTs) and found that field emission performance is optimal when the CNT height is comparable to the intertube spacing [54]. The field enhancement factor and FE characteristics of individual emitters can be investigated using scanning anode field emission mapping by adjusting the diameter, pitch, and length of the carbon nanofiber emitter arrays. The field enhancement factor is primarily governed by the aspect ratio of emitter height to radius. Maximum field enhancement factors are attained when the emitter spacing within the array is no less than twice their height [55]. The pitch and diameter of the circular CNT emitters were optimized to improve uniformity and achieve high field emission current. The CNT emitter array with a pitch of 120 µm and a diameter of 50 µm exhibited the optimal emission characteristics and achieved an optimal combination of the emission area and edge effects [56].

As the core component of the electron gun, the cathode directly determines the performance of CNT-based cold cathode electron guns. Many methods have been developed for CNT-based emitters as cathodes. Moreover, the performance of the electron gun is realized systematically through its various components. Researchers have studied the design and development of CNT cold cathode electron guns through different approaches, including electron beam focusing quality, electron transmittance, miniaturization, and electron beam modulation. The divergence of ejected electrons must be precisely controlled to form a specific-shaped electron beam in an electron gun for various applications. Adding the focusing structures controls the electron beam from the cathode composed of CNTs paste [57]. To effectively focus the emitted electron beams, the focusing electrode is concentrically surrounded by each gate hole in the gated field emitter arrays made of photosensitive CNT-containing paste [58]. In addition, electron-beam focusing in the devices using vertical CNT film as emitters can also be achieved by a self-focusing technique without additional focusing electrodes [59,60]. In order to improve the electron transmittance rate and reduce interception by the grid, some fabrication techniques of cathode-extractor assembly were designed. The electron transparency of 99% is obtained by processing the cathode substrate into a square column array congruent with the grid mesh [61]. The designed grid structure combines coarse and fine grids to create a uniform electric field distribution around it. Simulations show that this structure effectively increases electron transmission efficiency in the electron gun. Additionally, electron transmission efficiency results obtained using different grid aperture ratios were presented in an aligned CNT-film cold cathode electron gun [62]. The process of assembly alignment can also affect the performance of the electron gun with a CNT film cathode [63]. A miniaturized radio-frequency excited grid-free FE electron source based on the imprinted CNTs was investigated for high-frequency electronic devices [64]. Given the requirements of modulated electron beams for vacuum electron microwave sources, microwave electron guns designed using disordered CNTs and arrays of aligned CNTs as cold cathodes, respectively, have been developed [20,65].

Overall, there have been many theoretical and experimental studies to fabricate and explore good CNT-based electron guns. Indeed, these studies have been achieved based on specific morphologies of CNT cathode structures through controlling fabrication processes, nanotube orientations, surface structures, and composite materials. The morphological characteristics of CNT emitters directly govern their emission properties. The integration of these designed cathodes into practical electron guns and their resulting performance are analyzed for specific applications. A diverse set of cold cathode electron guns based on the CNTs has been studied and designed through different approaches. The review mainly introduces the research on CNT-based cold cathode electron guns through sorting out different morphology types of CNT emitters and by simulation. Finally, the work seeks to identify the existing challenges and proposes future development suggestions in the field, aiming to serve as a comprehensive reference for subsequent studies on CNT-based cold cathode electron guns.

## 2. Cold Electron Gun Based on Disordered CNTs Film Emitter

The disordered CNTs can be grown directly on various substrates, such as SS304 or silicon substrate, as a cathode in the cold cathode electron gun without the need for post-growth processing. Electrons can be emitted from the sidewall and the tip of individual CNT [66,67]. The disordered CNTs are bent and twisted together, which leads to a large number of randomly distributed sidewalls and tips. The morphology characteristic of the disordered CNT cathode surface can contribute to emitting electrons relatively uniformly. The shape of the disordered CNTs cathode can be complex shapes, such as truncated-cone [68] or striped-pattern [69], which enhances compatibility with equipment, and provides high flexibility in practical applications.

**Figure 1:**
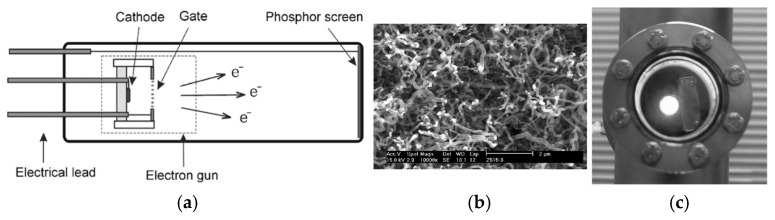
(**a**) The structure of the luminescent tube; (**b**) Scanning electron microscopy (SEM) image of CNTs; (**c**) The picture shows the luminescent tube operating [70].

Jun Chen et al. designed and fabricated the cold cathode electron gun with a coaxial structure based on CNT film [70], shown in Figure 1. The growing CNTs are twisted and wound together. The CNT film cathode was assembled into the luminescent tube, as schematically illustrated in Figure 1. The cathode current was regulated by an extraction gate. The image of the luminescent tube operating in the vacuum chamber was recorded via a CCD camera. Uniform high brightness was achieved using the cold cathode electron gun as a light-emitting tube. The maximum recorded luminance, measured by a photometer, reached over 10,000 cd/m^2^. And the direct current power consumption of the luminescent tube with a 0.1 mm gap between the gate and cathode is 1.12 W.

**Figure 2:**
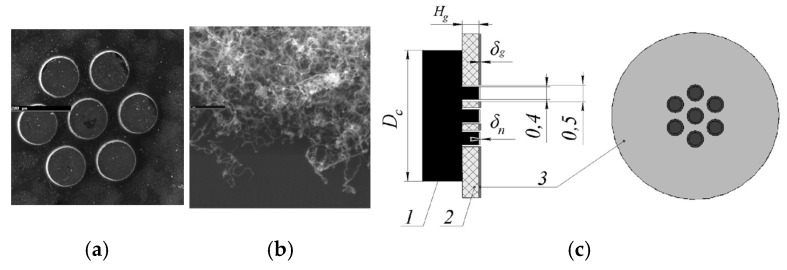
(**a**) SEM images of a 7-beam field-emission cathode and (**b**) CNTs; (**c**) The scheme of cathode-gate unit: 1—cathode; 2—dielectric plate; 3—gate-control electrode [71].

N. Bushuev et al. present a multibeam field-emission cathode with cylindrical projections coated with a thin film of randomly aligned CNTs [71], as shown in Figure 2. The thickness of the insulating substrate matches the projection height, and the emission current is not intercepted by the control gate deposited on the dielectric plate. The diameter of the cathode Dc is 3 mm. The experimental emission current can reach 25 mA with a 99% current flow pass rate, corresponding to a current density of 2.8 A/cm^2^.

**Figure 3:**
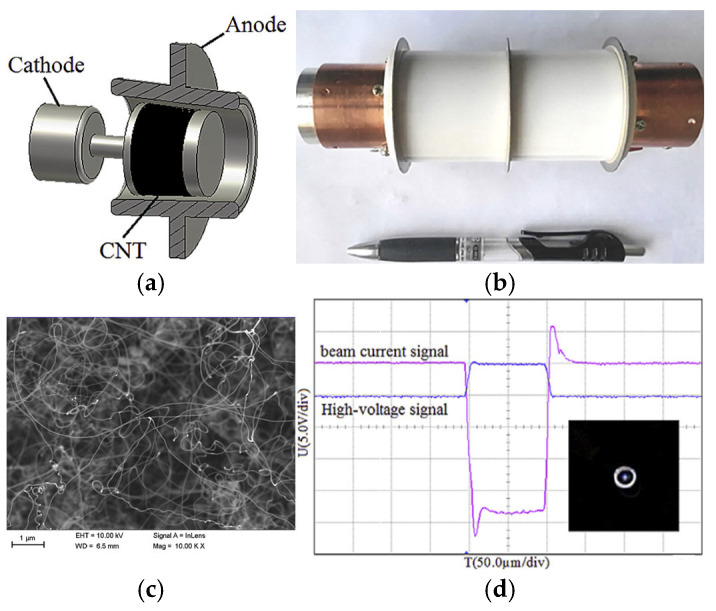
(**a**) Scheme of the truncated-cone CNT cold cathode coaxial diode; (**b**) Photo of the truncated-cone CNT cold cathode gun. (**c**) SEM micrograph of the typical as-grown CNT. (**d**) The test curve of high-voltage and beam current signal on oscilloscope, inset: beam spot on ITO-coated glass [68].

Xuesong Yuan et al. reported a gridded cold cathode electron gun based on disordered CNTs, which employs both an electrostatic field and a permanent magnetic field [72]. The inner and outer radii of the CNT cold cathode are 4.5 and 6.5 mm, respectively. The maximum electron beam current of 4 mA and the current transmission ratio of 58.8% are obtained in the experiment. The area compression ratio of the electron beam is about 10. They further prepared a truncated-cone CNT cold-cathode electron gun for FE vacuum electron radiation sources [68], as shown in Figure 3. The disordered CNT cold cathode is sandwiched between two stainless steel truncated cones. The cathode and anode were isolated by a 1 mm thick ceramic spacer (Figure 3b). In the experimental results, a maximum electron beam current of 50 mA at 35 kV was obtained. The average radius of the electron beam was about 1.1 mm with an area compression ratio of approximately 100 in a 9.2 T superconducting magnetic system. The proposed truncated-cone architecture electron gun, with the advantage of a high compression ratio, can be used to realize a gyrotron radiation source [73].

**Figure 4:**
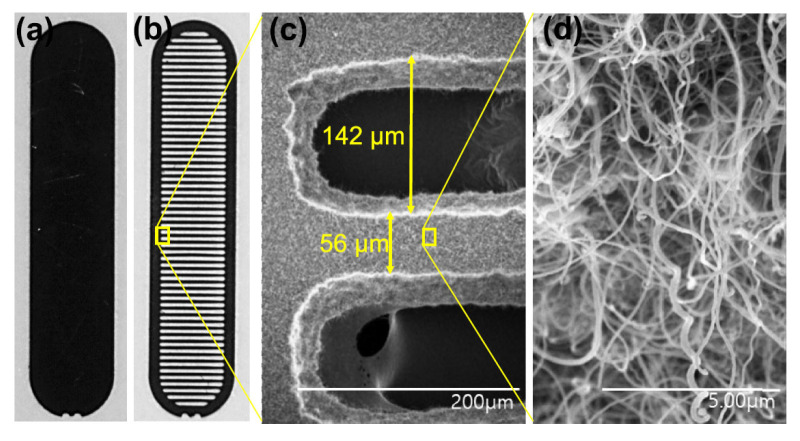
Optical picture of the fabricated (**a**) unpatterned and (**b**) stripe-patterned emitters; (**c**,**d**) SEM images of the stripe-patterned emitter on the alloy substrate [69].

**Figure 5:**
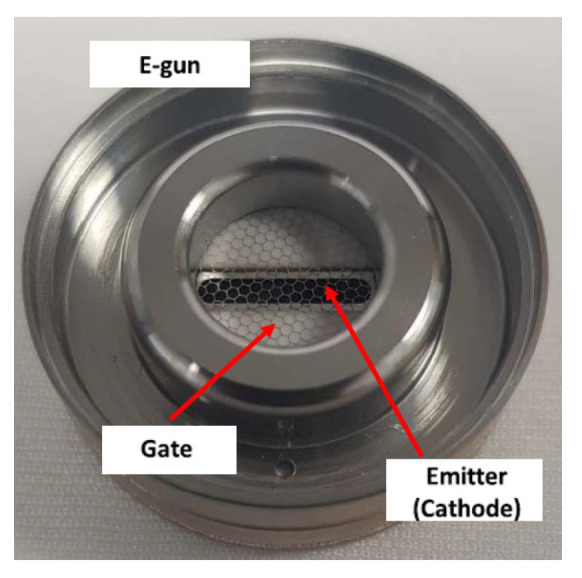
Electron gun with stripe-patterned emitter [69].

Xinghui Li et al. designed an electron gun with a nongated CNT cathode to overcome the high interception rate and power consumption on the metal mesh [74]. The randomly directed CNT cathode has a diameter of 0.6 mm and a curvature radius of 1.04 mm on the convex cathode surface. The electron gun with the nongated CNT cathode was employed in a magnetic-focused X-band traveling wave tube (TWT). The DC mode test results show that about 86% of the emission electrons can reach the collector in the TWT. Jongmin Lim et al. developed an electron gun with a cathode made of CNTs directly grown on a stripe-patterned alloy substrate [69], shown in Figure 4 and Figure 5. The CNTs exhibit a spaghetti-like orientation. The electron gun is of a triode type with a hexagonal gate electrode structure. The CNT emitters were installed in the electron gun, which comprises a cathode-gate structure and a focusing electrode. The type of electron gun structure can improve operational stability and field emission performance. Owing to the reduced screening effect and enhanced edge effect, the stripe-patterned emitter exhibits a higher FE performance and better stability compared to the unpatterned emitter. For example, the stripe-patterned emitter has an emission current of 2.32 mA, whereas the unpatterned emitter has only 0.22 mA under the grid voltage of 1.3 kV. Thus, the electron gun with the stripe-patterned emitter exhibits potential application in the next-generation X-ray technology.

**Figure 6:**
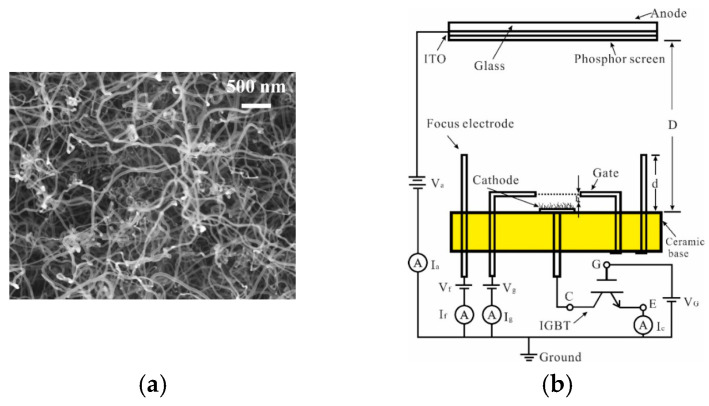

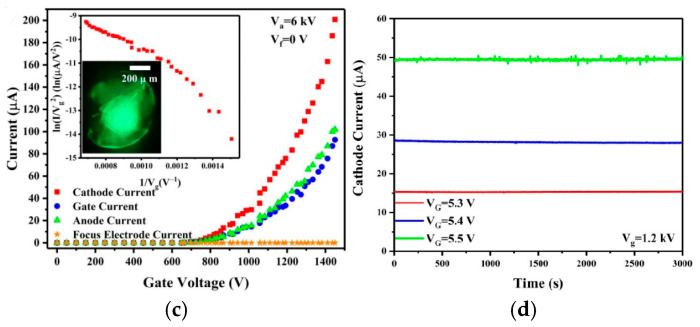
(**a**) SEM images of the prepared CNTs; (**b**) The scheme and measurement circuit of CNTs cold cathode electron gun; (**c**) FE I-V test characteristics of the CNT cold cathode electron gun (inset: corresponding F-N plot and image of beam emission acquired from phosphor screen; (**d**) The cathode emission current stability of CNT cold-cathode electron source with IGBT modulation [75].

**Figure 7:**
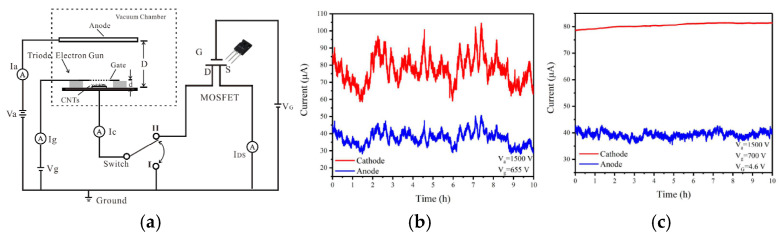
(**a**) The schematic of the structure and measurement set-up of the CNT cold-cathode electron gun connected in series with a metal-oxide-semiconductor field-effect transistor; The current stability over 10 h (**b**) in the CNT cold-cathode electron gun; (**c**) in the CNT cold-cathode electron gun connected in series with a MOSFET at a gate voltage of 4.6 V [76].

Yajie Guo et al. developed a gated cold cathode electron gun based on CNTs for an X-ray source [75], as shown in Figure 6. The fabricated CNTs are densely packed and disordered. The electron gun achieved a maximum current of 200 µA, corresponding to an emission current density of 9.55 mA/cm^2^ and a transmittance of 53%. Through insulated gate bipolar transistor (IGBT) modulation, the electron gun can achieve a highly stable cathode current with fluctuation of less than 0.5%. Further, they proposed a cold cathode triode electron gun with a disordered CNTs cathode driven by a metal-oxide-semiconductor field-effect transistor (MOSFET) [76], as shown in Figure 7. The cathode and anode current fluctuations in the CNT cold-cathode electron gun in series with a MOSFET were measured at 0.87% and 2.3%, respectively. Compared to the electron gun without a MOSFET, both the cathode and anode current stabilities were significantly improved. These results demonstrate that employing an active control component, such as a MOSFET, is an effective method for stabilizing field electron emission in CNT cold-cathode electron guns.

**Figure 8:**
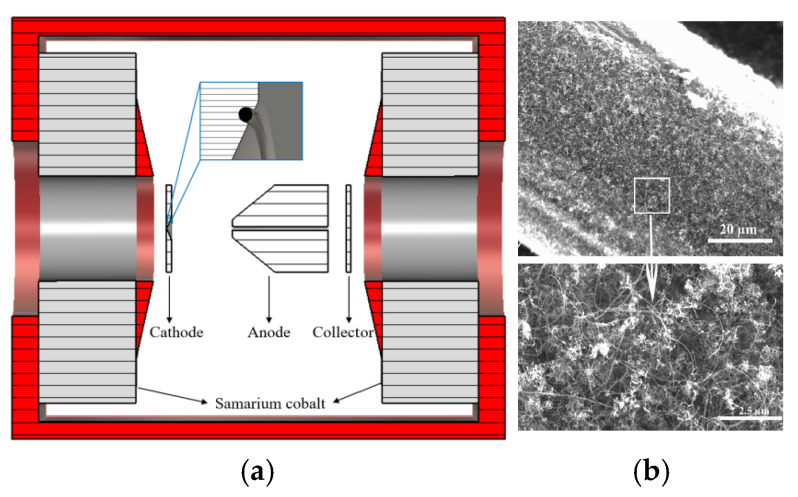
(**a**) Schematic illustration of CNT-based ring cathode electron gun [77]. Inset: the Ni_80_Cr_20_ alloy wire; (**b**) SEM picture of CNTs grown on Ni_80_Cr_20_ alloy wire, along with top-surface SEM picture of CNT emitter [78].

Yifan Zu et al. developed an annular beam CNT cold cathode electron gun [77]. The gun is based on a Ni_80_Cr_20_ alloy-supported CNT emitter and is intended for applications in the emerging field of terahertz (THz) vacuum microelectronic radiation sources [77,78], as shown in Figure 8. The emitter is fabricated by connecting the CNT film emitter end-to-end. It was then inserted in the rectangular groove formed in the inclined plane. Through parametrically optimized macro-scale simulation, the electron gun emitted up to a total of 14.8 kV/85.8 mA of annular electron beam, with a high beam current density of 156 A/cm^2^.

## 3. Cold Electron Gun Based on Aligned CNTs Emitter

The vertically aligned CNTs cathode has an ordered arrangement and unique geometry characteristics, with their tips directly exposed to an electric field, allowing for more effective concentration of the electric field. Vertically-aligned CNTs are promising emitters in cold cathode vacuum electronics, which have significant advantages in electron emission characteristics, such as low turn-on electric field, low threshold electric field, and efficient performance at low operating voltages [79]. An individually aligned CNT emitter also demonstrates superior mechanical robustness and thermal conductivity, thereby facilitating sustained electron emission and enhancing the operational reliability and stability of the electron gun. The aligned CNTs can be grown on various substrates and easily made to create precisely patterned arrays. In addition, the growth process of the aligned CNT emitter can be compatible with the microfabrication process and easily integrated with other micro/nano devices [60].

### 3.1. Cold Electron Gun Based on Single-Aligned CNTs Bundle Emitter

**Figure 9:**
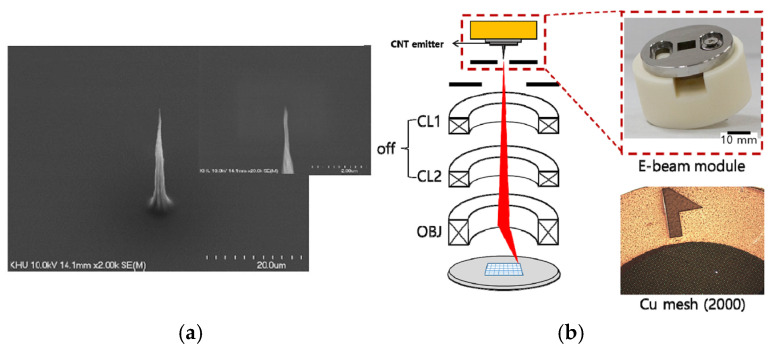
(**a**) Initial CNT cold cathode; (**b**) Schematic of the electron beam module and the secondary electron image system and Cu mesh object [8].

**Figure 10:**
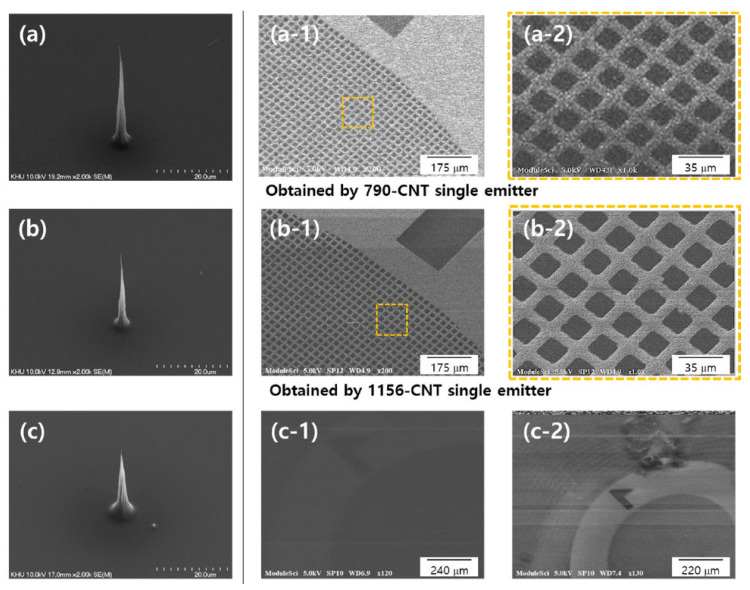
SEM images acquired using CNT cold cathodes with different geometric factor and relative extraction voltage (ΔV) values of (**a**) 790, 1200 V; (**b**) 1560, 900 V; (**c**) 417, 2100 V; (**a-1**–**c-1**) SEM images of Cu grid mesh acquired at an acceleration voltage of 5 kV and an emission current of 1 µA; (**a-2**) A 1000× magnification of the dashed square region in (**a-1**); (**b-2**) A 1000× magnification of the dashed square area in (**b-1**); (**c-2**) SEM image obtained at 5 kV and 3 µA emission current [80].

An aligned CNT emitter can also be grown on a large area and be conveniently shaped into surface morphologies as required. The CNT emitter can also be handily transferred from these growth substrates to the cathode pedestal of an electron gun. A single aligned CNT emitter can achieve high brightness and therefore can serve as a small electron emission source. The effects of the macroscopic cathode structure of a single aligned CNT bundle have also been investigated. Ha Rim Lee et al. fabricated a novel cold cathode electron gun based on vertically full-aligned CNTs and studied the beam properties, followed by imaging of Cu mesh for electron microscope application [8], as shown in Figure 9. The electron beam indicated a resolution of 3.5 µm without a condenser lens, which would be used for low-cost moderate resolution imaging. Further, they systematically investigated the influence of structural properties of CNT cold cathodes with different geometric factor values on the beam characteristics and secondary electron imaging resolution [80]. The CNT cold cathode with a geometric factor of 1560 generated a focused beam with high brightness and small beam diameter, consequently enabling the acquisition of high-resolution SEM images, compared with cathodes with the geometric factor values of 790 and 417, as shown in Figure 10.

**Figure 11:**
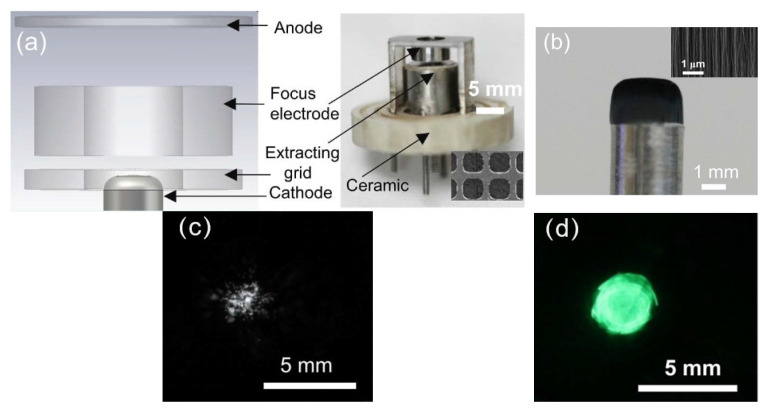
(**a**) Schematic of the cold electron gun structure and the picture of the assembled electron gun. Inset: SEM image of the Mo grid; (**b**) Optical photograph of the convex CNT emitter cathode. Inset: SEM image of the CNT emitter; (**c**) The sites distribution of the convex CNT emitter on the indium tin oxide glass; (**d**) Electron beam spot patterns of the electron gun on the phosphor screen [81].

FE uniformity of the CNT cathode can be greatly affected by the edge effect, and the laminar flow of the cold cathode electron gun can also be disturbed. Jiupeng Li et al. fabricated a convex cold cathode electron gun based on vertically aligned multiwalled CNTs [81], as shown in Figure 11. The convex CNT emitter with a curvature radius was installed in the electron gun as the cathode. The majority of the emission sites were evenly distributed across the central region. The brightness of the beam spot exhibits relatively uniform brightness with no branching. The convex-shaped CNT emitter cathode can improve the site distribution uniformity by greatly reducing the edge effect. The electron beam focusing and laminar flow of the cold cathode gun have also been improved. The electron gun with macroscopic cathode structure has important potential applications in the vacuum microwave device radiation source [82].

Zhaoying Xu et al. developed a high-current arcing in-situ treatment method to reshape the top morphology of aligned CNT pillars, thereby improving the performance in electron transparency and emission uniformity [83]. Lizhou Wang et al. proposed a CNT cold cathode electron gun with a coplanar quadrupole focusing structure to realize a multifunctional electron beam shape tailoring [84]. Research showed that the electron beam spot area compression ratio of the electron beam was 34.6. A circular electron beam can be transformed into a sheet-like beam. The beam spot position shifting in both X- and Y-axis within a distance can be realized. Junbo Ren et al. presented a sheet-shaped CNT cold-cathode electron gun with an asymmetric focus electrode [85]. The asymmetric focusing electrode can independently control the focal point in two directions, resulting in a unified sheet electron beam focusing. The sheet-beam electron gun can be applied to a backward wave oscillator [86].

**Figure 12:**
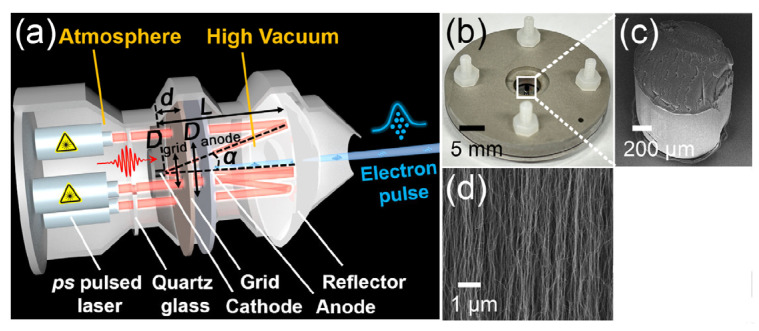
(**a**) Scheme of the electron gun layout; (**b**) Picture of the assembled cathode-grid-anode component; (**c**,**d**) SEM images of the CNT cathode [87].

**Figure 13:**
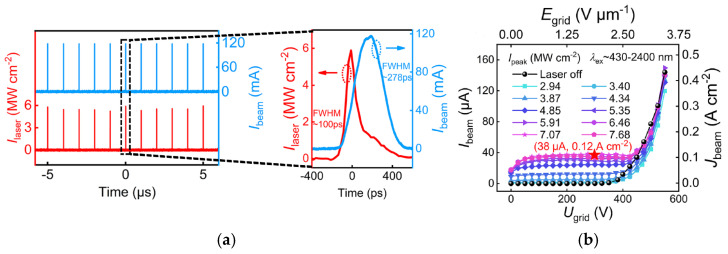
(**a**) The time-domain waveform of the electron beam current (I_beam_) pulses and the excited ps laser intensity (I_laser_) pulses; (**b**) I_beam_ and J_beam_ versus cathode-grid voltages (U_grid_) and cathode-grid electric field (E_grid)_ curves under ps laser excitation [87].

Dong Han et al. proposed a CNT cold-cathode electron gun by a photo-electric synergistic excitation, which can be used for ultrafast electron characterization and high-frequency radiation sources [87], as shown in Figure 12. The gun is composed of two parts, specifically for FE emission and photoexcitation. The excitation threshold is effectively decreased by the photoelectric co-excited device. The electron gun is capable of generating ultrafast and ultrashort pulsed electron beams with a repetition frequency of 1 MHz and a pulse width of 278 ps. At a peak I_beam_ of 120 mA, the current pulses exhibit a certain degree of delay and spreading to the laser pulses, as shown in Figure 13. The studies on the emission characteristics and mechanisms of the electron gun show that the pure FE I_beam_ characteristics of CNTs are comparable to the I_beam_ under photo-electric co-excitation. The role of the ps laser is manifested mainly when the electrostatic field is weak. Ke Chen et al. have achieved a single-tube CNT-based electron source with a low energy dispersion (~0.3 eV) and a short pulse width (~13 fs) for ultrafast electron microscopy [88].

### 3.2. Cold Electron Gun Based on Aligned CNTs Arrays Emitter

The final current that can be utilized is the current that can reach the anode. The fabrication of CNT arrays provides an effective approach for eliminating the electrostatic screening effect and reducing the leakage current ratio through the electron extraction gate [89]. The structure and material properties of the electron extraction electrode can also significantly affect the electron beam current [90]. Usually, the electron extraction electrode is fabricated where the top of the CNT arrays cold cathode is aligned with the mesh of the gate electrode. Different arrangements of the aligned CNTs arrays cathodes have a significant impact on the distribution of the electric field on the cathode surface, which can enhance FE stability [91].

**Figure 14:**
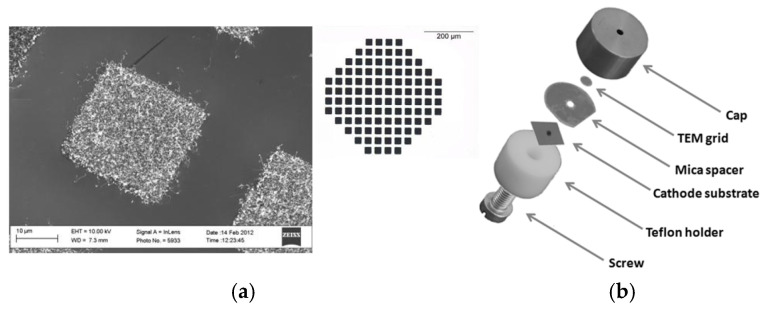
(**a**) SEM image of CNTs pattern; inset: optical microscope image of CNTs patterns; (**b**) Picture of the realized assembly parts [63].

Giacomo Ulisse et al. reported a CNT cold-cathode electron gun assembled with an external extracting grid and an electrostatic focusing [63], as shown in Figure 14. The CNT array was patterned in a circular form to act as an emitter. The CNT cathode gun with an external grid was fabricated through high-precision mechanical processing. The measurements on the assembled electron gun were performed under two conditions: misalignment and perfect alignment between the emitting area and the extracting grid. They demonstrated that the assembly process could greatly affect the current density and electron transparency ratio between the anode current and emitted current.

**Figure 15:**
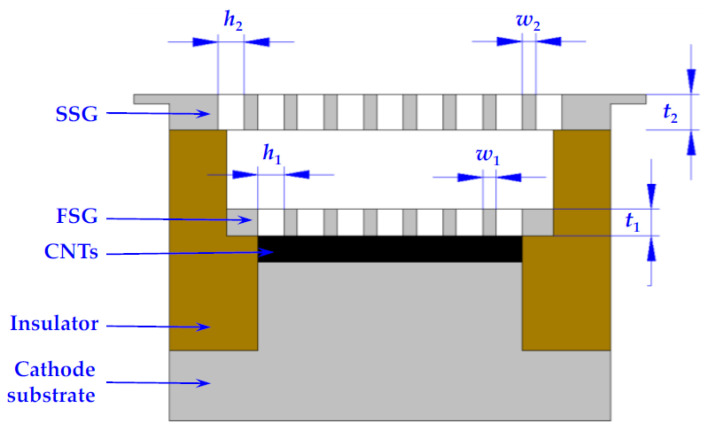
Schematic illustration of the dual-gridded FE structure based on a CNT cold cathode. FSG: first separating grid. SSG: second separating grid [92].

**Figure 16:**
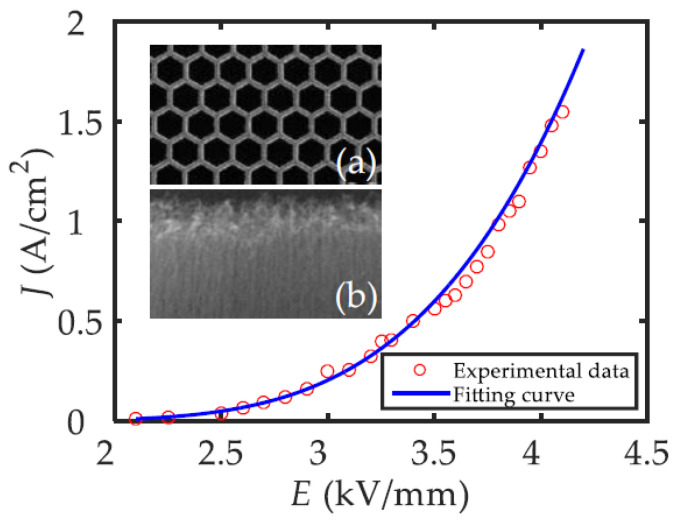
Experimental and fitting FE current density profiles as a function of applied electric field. Insets: (**a**) Top-view SEM micrograph of the honeycomb mesh; (**b**) Cross-sectional SEM micrograph of the aligned CNT array [92].

**Figure 17:**
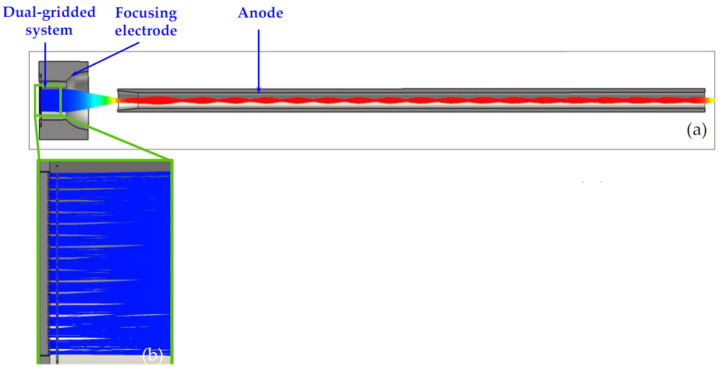
(**a**) Simulated electron beam trajectories in the dual-gridded, CNT-based electron gun; (**b**) Expanded dual-gridded system view, with no electron interception observed [92].

Qingyun Chen et al. proposed a novel dual-gridded cold cathode electron gun based on a vertically aligned CNT cold cathode [92]. The CNT cathode array was shaped by the first separating grid attached to the CNT cathode surface. The second separating grid was used to control the extraction of electrons from the CNT emitters. The two grids, which are coaxially connected by the insulator, aim to address the non-uniformity of the electric field distribution. The electron gun with a dual-gridded structure can greatly reduce electrostatic screening and edge effects, improving the cathode surface electric field distribution, as shown in Figure 15. The FE properties and SEM image of the CNT emitters are shown in Figure 16. The CNTs were synthesized into a network with hexagonal pores. The maximum operating emission current density is 1.5 A/cm^2^, corresponding to an electric field of 4.17 kV/mm. The construction and beam trajectories of the electron gun are shown in Figure 17. The electron beam is focused by the electric and magnetic fields with a high-compression ratio of 39. The electron beam transparency can be achieved 100% theoretically.

**Figure 18:**
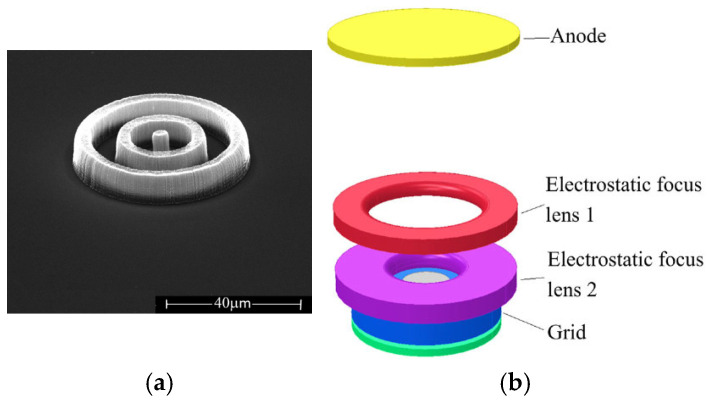
(**a**) SEM image of the overall shape of the CNT array; (**b**) Schematic model of electron gun structure [93].

Xiaobing Li et al. fabricated a cold cathode electron gun based on a vertically-aligned ring-shaped CNT emitter for a full vacuum-sealed macrofocus X-ray tube [93], as shown in Figure 18. The electron gun, consisting of an external grid gate and two ring-shaped focusing electrodes, can control electron beam convergence. The electron gun can obtain an emission current of 200 µA, with a corresponding maximum current density greater than 10 A/cm^2^. The microfocus X-ray tube with very high spatial resolution has reached the leading level in its field, which can be used in clearly distinguishing JIMA card, chips, and multilayer printed circuit board [93,94].

**Figure 19:**
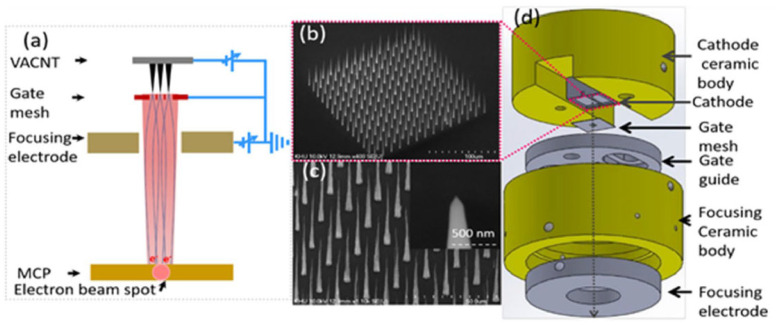
(**a**) The schematic diagram of the power supply and electron beam imaging process for 14 × 14 CNT emitters in a microchannel plate; (**b**,**c**) CNT emitter SEM images. Inset in (**c**): The individual vertically aligned CNT emitter; (**d**) The electron beam alignment and module design for solid work [96].

Bishwa Chandra Adhikari et al. prepared a single electron beam source using vertically aligned cone-shaped CNTs with 14 × 14 emitters. The microchannel plate made of an electrically insulating material containing a hexagonal array of tiny holes is used to capture FE electron beam spots, which can mitigate the signal noise effect and reduce damage to CNT tips. The uniform FE microscopy image can be acquired by adjusting the exposure time and applied voltage, which can be used to investigate beam trajectories and the beam spot sizes [95]. Furthermore, Bishwa Chandra Adhikari et al. developed a cold cathode electron gun based on multi-walled CNTs, as shown in Figure 19 [96]. An electron beam from this CNT cold cathode was simulated with different focusing schemes. The minimum beam spot size of the CNT emitters is calculated to be 0.9 mm within the microchannel plate, and the beam current density is enhanced by 8 times compared to the non-focused scheme.

### 3.3. Aligned CNTs Cold Cathode Electron Gun Based on Micro-Fabrication Technique

**Figure 20:**
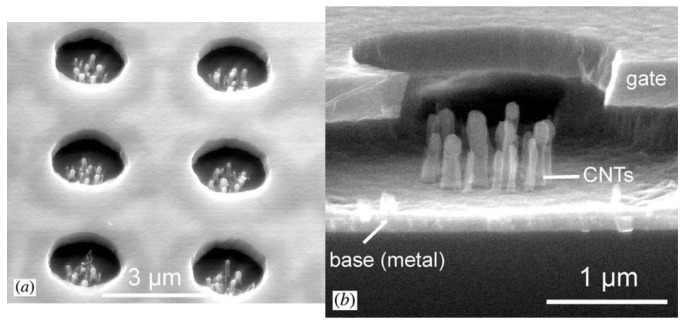
(**a**) Tilted (45°) top SEM image of the microcathode; (**b**) Cross-sectional SEM image of a microcathode [97].

**Figure 21:**
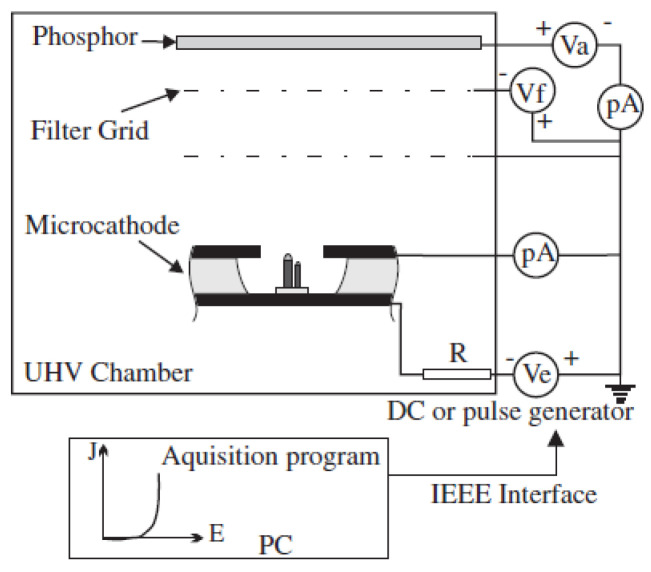
Schematic diagram of the measurement system [97].

The aligned CNT emitters fabricated by microfabrication methods, serving as a cold cathode, can facilitate miniaturization, high efficiency, and low energy consumption in various devices, particularly in high-frequency microwave and portable devices. A single CNT emitter per gate can also avoid the screening effect among the nanotubes. G. Pirio et al. fabricated an FE source using microcathodes based on vertically aligned CNTs for vacuum microelectronic devices, as shown in Figure 20 and Figure 21 [97]. The microcathodes were made by a self-aligned fabrication process, which contains a patterned array of 150 × 150 holes with a diameter of 1 µm at a pitch of 4 µm. Using the cathode, a peak current density of 0.6 mA cm^−2^ was obtained at 40 V, with a duty cycle of 0.5%.

**Figure 22:**
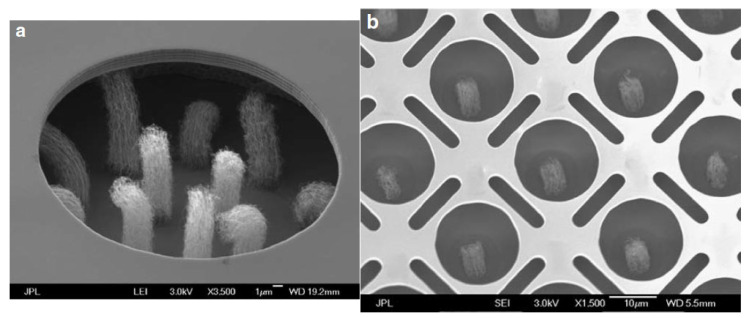
Monolithically gate-integrated CNT field emitters. (**a**) SEM image of multi-bundle design (25 µm diameter) gate; (**b**) SEM image of a single-bundle design (20 µm diameter gate) [98].

Manohara. H.M. et al. designed and fabricated a CNT field emitter by the monolithic electrode integration technique using a double silicon on insulator process [98], which enables multiple-level electrode integration to realize a miniature electron gun for THz vacuum tube sources, as shown in Figure 22. The integrated gate electrode overhangs near the tips of the CNT bundle array with only a few microns in lateral separation. In the triode configuration, with the anode positioned at a separation of approximately 460 µm, the measured electron emission efficiency was 70–80%. The lifetime of these cathodes would be affected by the failure modes of a gradual decay of emission and dislodgement of CNT bundles.

**Figure 23:**
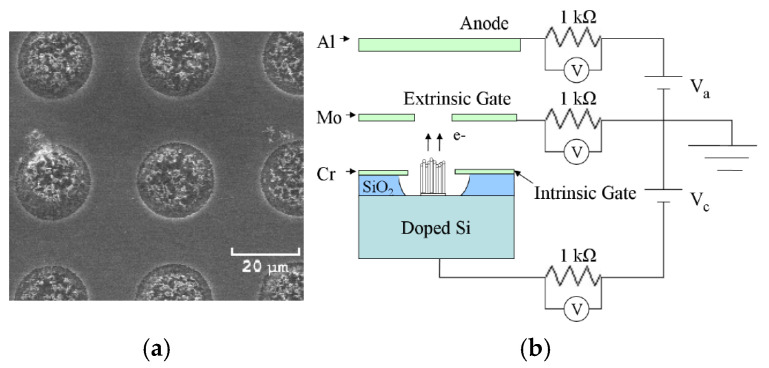
(**a**) Micrographs of the CNT arrays; (**b**) Electrical schematic of triode configuration [100].

Logan T. Williams et al. fabricated CNT cathodes based on vertically aligned CNT arrays for applications of small satellites and neutralization of exhaust plumes of electric propulsion devices [99,100], shown in Figure 23. The CNT arrays are completely recessed inside the insulator. An external gate is suspended above the wafer surface to generate an electric field that drives electron emission downstream of the cathode. This setup adopts a triode configuration connecting the cathode, external gate, and anode. The measured maximum current density is 9.08 mA/cm^2,^ and the maximum life span is 368 h. In addition, they also studied the behavior of cathode current emission, consisting of oscillations and sudden shifts. These observations were potentially attributed to the presence of water vapor. The primary cause of cathode failure is ohmic heating from oxidative ablation at the root of the nanotube and field evaporation at the tip [101].

**Figure 24:**
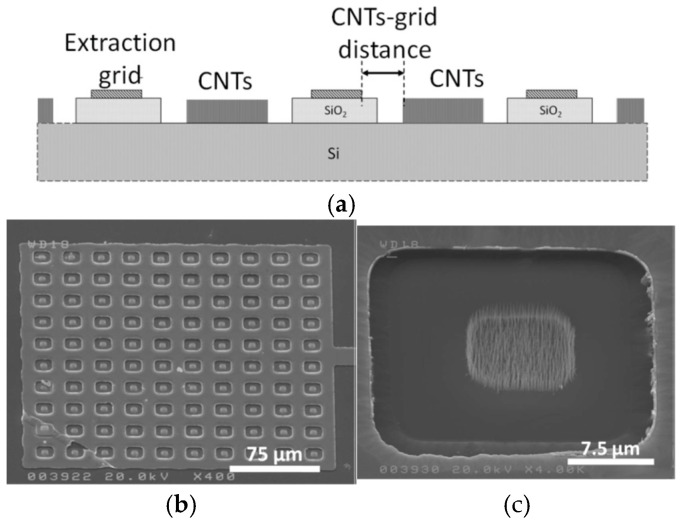
(**a**) Configuration of cathode with the integrated grid; (**b**) The cathode with an array of 10 × 10 emitting zones; (**c**) SEM image of one CNT emitting zone [102].

Giacomo Ulisse et al. reported an aligned CNT array-based cold cathode electron gun with an integrated grid structure [102], as shown in Figure 24. Another CNT cold cathode electron gun with an external extracting grid structure was used for comparison. The electron gun with an external extraction grid showed a maximum current density of 3.2 mA/cm^2^ in a field of 17 V/µm and a transparency of 72%. The electron gun with integrated grid showed a maximum current density of 74 mA/cm^2^ in an applied field of 11 V/µm and a transparency of 75%, which represents a more suitable structure for low-power consumption integrated vacuum electronics compared to the external grid structure.

**Figure 25:**
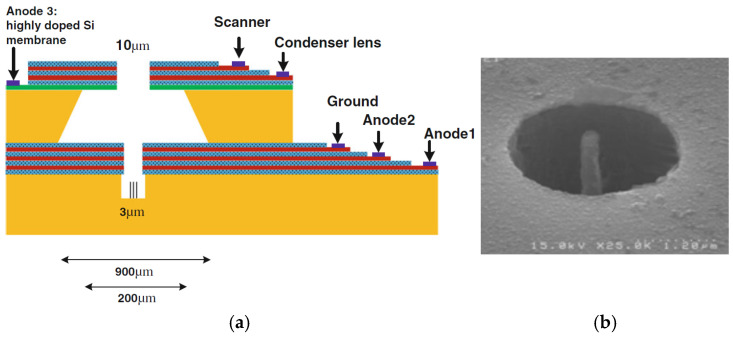
(**a**) The schematic drawing for the miniaturized electron gun using one accelerating cap; (**b**) SEM image of vertical CNT grown in the center of the hole as an emitter [103].

Javad Koohsorkhi et al. designed and fabricated a miniaturized electron gun based on vertically aligned CNTs [103], as shown in Figure 25. The electron gun consists of two parts: the first part consists of an electron source, extracting and focusing electrodes, and the second part contains a condenser lens, lens aperture, objective lens, and scanning electrodes. Vertical CNT at the center of the micro-sized hole was used as an emitter. The average current emitted from each CNT emitter is approximately 3.5 nA under an electric field of 1 V/µm. The simulation and experimental results show the good controllability of electron beam shape and energy distribution of the electrons by applying a proper voltage to the anodes and objective lens, making the miniaturized electron guns suitable for low-energy electron microscopy and mini-SEM units. Ha Rim Lee et al. fabricated a triode electron gun cold cathode based on vertically aligned CNTs, which can generate high-resolution electron beams [104]. The gate hole in size is 54.5% of the cathode area. The cold cathode has 9 CNT emitters with an emission current of more than 60 µA at 5.1 V/µm. The transmission ratio of the 14 µA electron beam current reaches 77%. The phosphor lighting image with the nine emitters was observed at an emission current of 3 µA and 5 kV acceleration voltage. The electron beam source with high resolution could be applied to various microscope systems.

## 4. Cold Cathode Electron Gun Based on CNTs Paste Emitter

Some CNT emitters are prepared by imprinting CNT pastes. The CNT pastes consist of CNTs and other ingredients in proportion, such as organic binders and silver powder. The performances of the emitters are related to the compositions, contents, and post-treatment processes of the pastes. The CNT paste emitter is currently one of the most promising CNT FE emitters [105], with advantages of simple operation, short preparation cycle, low cost, large-area film formation, and mass production. Additionally, the CNT emitter can be integrated not only with rigid substrates but also with flexible ones. The field electron emissions of CNT paste-emitters still need to be fully understood and optimally designed for specific electron source applications [106].

**Figure 26:**
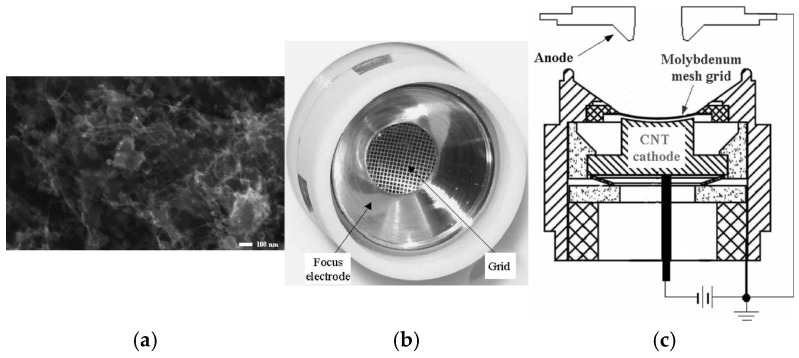
(**a**) SEM image of the CNTs paste; (**b**) Picture of gridded CNT cold-cathode electron gun; (**c**) Experimental configuration of a diode-type FE performance test [108].

**Figure 27:**
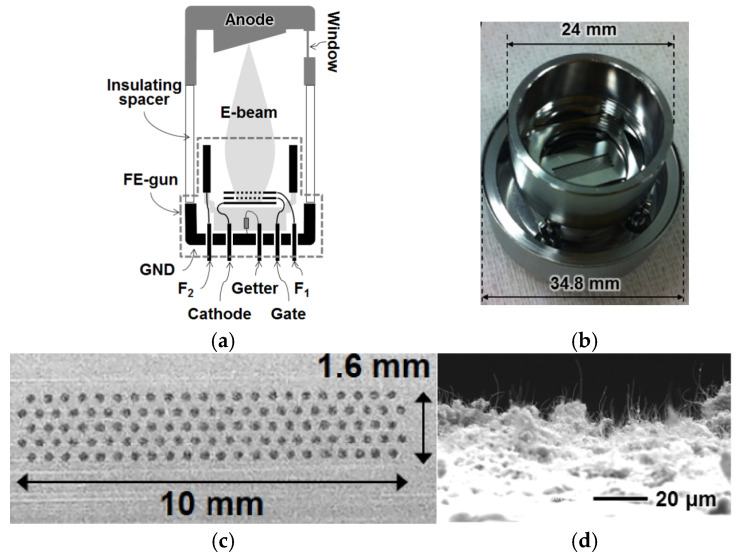
(**a**) Schematic of the FE gun and the vacuum-sealed X-ray tube; (**b**) Picture of the field-emission electron gun module; (**c**) Optical and (**d**) SEM images of the CNTs paste emitters on the Kovar cathode substrate in the electron gun module [57].

Shaozhi Deng et al. studied the frequency characteristics of the luminescent element using the CNT-based composite as a cold-cathode [107]. There are two delay times (on the rising edge and falling edge) between the input pulse and the corresponding output pulse. As the frequency of the input pulse increases, the amplitude of the output pulse may decrease in the high-frequency range. Furthermore, the dependence of the cutoff frequency on the structural parameters of the electron gun of the lighting element has also been found. Hae Jin Kim et al. designed a double-gridded focusing gun with a curved CNT cold cathode for traveling-wave tube applications [108], as shown in Figure 26. The CNT cathode made of a well-dispersed CNT paste was printed onto a designed cathode with a diameter and spherical radius of 4.3 and 9.73 mm, respectively. The electron gun performance of the CNT paste cathode was studied, and a pulsed anode current of 1.8 mA and an electron beam current transmission ratio of 64% were measured. Jin-Woo Jeong et al. designed and fabricated a field-emission electron gun consisting of a CNT emitter cathode, gate, focusing electrode 1 (F1), and 2 (F2) for the X-ray tube [57], as shown in Figure 27. The CNT paste is composed of multi-walled CNTs, organic powder of ethyl cellulose, inorganic fillers of Cu alloy, and Al_2_O_3_ particles. The design focusing structure consisting of F1 and F2 has a micromesh and a macrocylinder, which leads to more focused electron beams on the anode target when compared with the two macrolens [109,110]. When the cathode current attained 50 mA, a small focal spot of around 0.3 mm was generated on the anode target. Furthermore, in order to obtain a large focusing capability, Jae-Woo Kim et al. fabricated an electrostatic focusing lens (FL) consisting of a micro-FL with plenty of holes near the gate and a macro-FL with a single aperture. When the FL is controlled across a broad range of gate and anode voltages, the focused spot is maintained [111].

**Figure 28:**
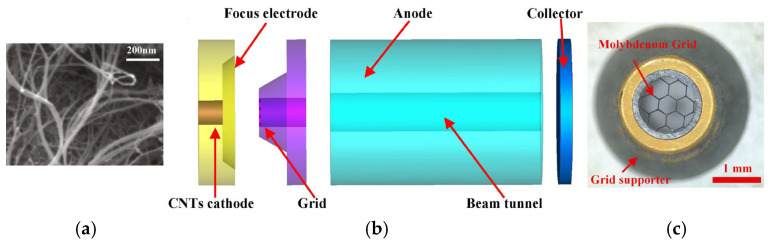
(**a**) SEM image of the printed CNT film; (**b**) Schematic of the electron gun based on CNT cathode; (**c**) Picture of the grid structure in the electron gun [112].

Jian Zhang et al. designed a CNT cold cathode electron gun with laminar flow features [112], as shown in Figure 28. The cathode is composed of randomly oriented printed CNTs on the surface. By designing a unique electric focusing lens, electrons are focused at low energies between the cathode and grid electrode to reduce electron trajectory crossing and improve the laminarity of the electron beam. The CNT electron gun is able to generate a maximum current of 9.76 mA, outputting a current of 8.08 mA, with a corresponding grid interception rate of less than 22%. The corresponding current density is 1.24 A/cm^2^, and the interception rate of the electrons through the grid electrode is less than 22%. The CNT cold cathode electron gun is very promising for use in TWT applications.

**Figure 29:**
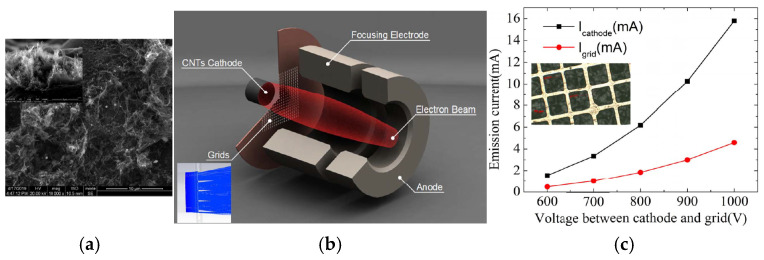
(**a**) SEM image of the CNT cathode. Inset: the side view of the CNT cathode; (**b**) Schematic of the grid-controlled cold electron gun. Inset: enlarged electron trajectories near the grid; (**c**) Simulated emission current (I_cathode_) and the grid current (I_grid_) of the electron gun with CNT cathode as a function of the voltage between cathode and grid. Inset: the microscope image of the Mo grid [113].

Ruirui Jiang et al. designed a cold cathode electron gun with a single-wall CNT cathode for the Ka-band TWT [113], as shown in Figure 29. The CNT cathode was made by the direct imprinting method [105]. The CNT cathode surface covers countless protrusions. The anode and focusing electrode consist of metal cylindrical structures with different holes. Electrons are drawn from the cathode surface under grid voltage. Forward-moving electrons undergo compression by the focusing electrode and are ultimately accelerated by the anode. The turn-on field of the CNT cathode is low, and the operating voltage is also low. The very low turn-on field of 0.57 MV/m could be realized, which can greatly reduce the operating voltage. The maximum emission current obtained was about 10 mA, corresponding to an emission current density of 1.4 A/cm^2^. The interception rate of electrons emitted in the electron gun simulation is approximately 30%. They further investigated a grid-free cold cathode electron gun based on embedded CNTs [114]. The CNT cold cathode is fabricated through the screen printing method with a silver paste buffer layer. The CNT cold cathode exhibits a turn-on field of 0.78 V/µm and a current density of 11 A/cm^2^, with its electron beam confined within a 0.05 mm radius by a magnet focusing. The electron gun is suitable for a miniaturized THz TWT.

## 5. Cold Cathode Electron Gun Based on Other Forms of CNT Emitters

In addition to the several cold cathode emitters based on CNTs introduced above, there are also other morphological types of CNT-based electron emitters, or CNTs combined with other field-emission electron emitters by some alternative manufacturing techniques. Field electron emission is significantly affected by the surface condition of emitters [115]. These morphological types of CNT emitters have various surface conditions, and some of them could provide some opportunities for specific application needs when used as cold cathodes of electron guns. For example, FE cathodes made from single CNT fibers have demonstrated high electrical conductivity, high emission currents, and long lifetimes, which show good potential for electron sources in vacuum electron devices [116,117].

**Figure 30:**
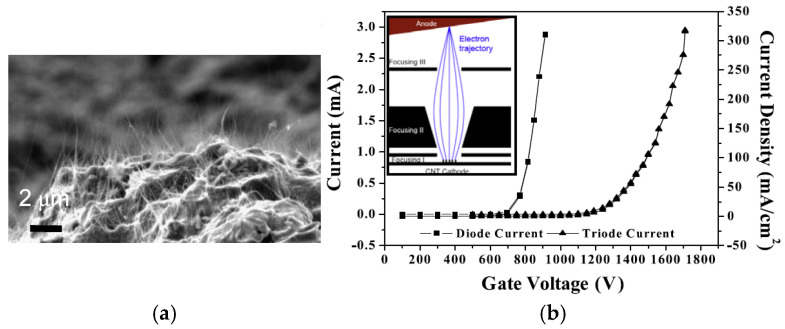
(**a**) Cross-sectional SEM image of the CNT cathode after the activation process; (**b**) FE current as a function of the applied gate voltage at constant anode voltage. For comparison, the data from the same cathode measured in the diode structure are also shown [123].

Chemical vapor deposition (CVD), paste printing, and electrophoresis represent several conventional methods for the preparation of CNTs. Among these methods for the development of CNTs, CVD is the primary method for growing CNTs and also an adapted method for large-scale production of CNTs at low cost [118]. The method offers several advantages, including maturity in industrial applications, scalability, adaptability, and compatibility with microfabrication processes [119,120]. However, it requires relatively high temperatures and faces persistent challenges in large-area fabrication. Paste printing technique can provide an efficient and low-cost solution for the large-area fabrication of the CNT arrays in industrial research [13]. The technique can achieve a resolution for a single patterned line on the order of tens of micrometers when combined with photolithography. The technique requires the use of binders and an activation treatment. The electrophoresis method is a widely used industrial process, characterized by its high throughput and automation. The method can enable the deposition of pre-fabricated CNTs on various substrate surfaces with controlled morphology and thickness ranging from tens of nanometers to several micrometers [121]. The electrophoretic deposition process is lower cost and can be readily scaled up to fabricate CNT. X. Calderón-Colón et al. used the electrophoresis method to prepare composite CNT films, optimizing the surface orientation and density of the CNTs. The vertically aligned CNTs on the substrate surface, combined with optimized morphological parameters, could improve the adhesion of CNTs to the substrate [122,123], as shown in Figure 30. The morphology of the CNT emitter obtained by the method is similar to that fabricated by imprinting technology. For CNT cathodes with long-term stability under high current and high voltage conditions, a stable cathode current of 3 mA (325 mA cm^−2^) and a transmittance of 60% were obtained from the 0.50 mm × 2.35 mm elliptical CNT cathode.

**Figure 31:**
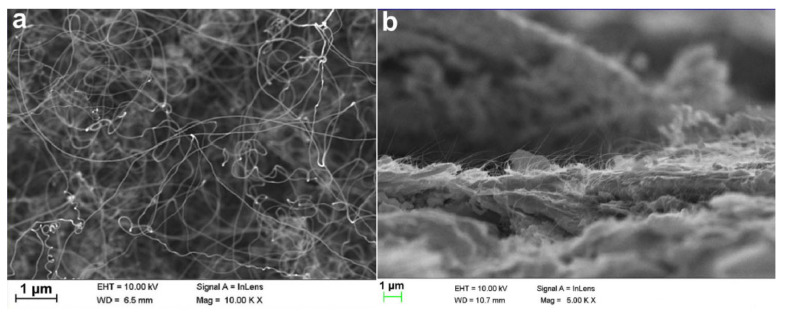
(**a**) SEM of an unoptimized misaligned and overly dense CNT cold cathode; (**b**) edge-view SEM of density and alignment optimized as-grown dense CNT forest thin film on a stainless steel substrate [124].

**Figure 32:**
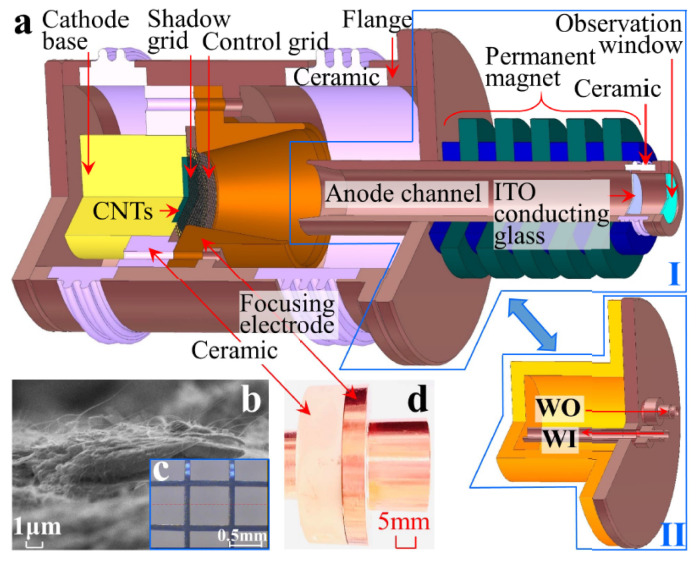
(**a**) Configuration of CNT cold cathode electron gun with two grids and anode block (water circulation system: water output-WO, water input-WI); (**b**) Edge-view SEM of an as-grown dense CNT forest thin film; (**c**) Optical micrograph of the grid; (**d**) Photograph of the dual-gridded assembly with the focusing electrode [125].

Xuesong Yuan et al. optimized the cathode nanomaterial by precisely controlling CNT growth to reduce the CNT packing density and increase the typically vertical alignment of CNTs on the emitter [124], as shown in Figure 31. CNTs grown on a truncated-cone SS304 stainless steel are used as cathodes, and the anode serves to extract electrons. Owing to reduced neighbor electrostatic shielding, a maximum emission current of 265.5 mA (current density of 188.3 mA/cm^2^) was observed. Then, they used the CNTs to make a truncated-cone magnetic injection CNT cold-cathode electron gun for a Ka-band backward wave oscillator. Qingyun Chen et al. further developed an improved dual-gridded cold cathode electron gun based on CNT forest thin film [125], as shown in Figure 32. The electron gun is equipped with two grids: one is a shadow grid attached to the surface of the CNT cathode, and the other is a control grid aligned with the shadow grid. The dual-gridded structure of the electron gun can increase electron transparency and has a relatively uniform field distribution. The designed structure of the electron gun is similar to their previous work. Herein, a maximum anode pulse current of 700 mA under an electric field of 8.1 kV/mm during pulse-mode processing has been realized. The electron beam transparency is up to 100%, and the corresponding compression ratio is 19.

**Figure 33:**
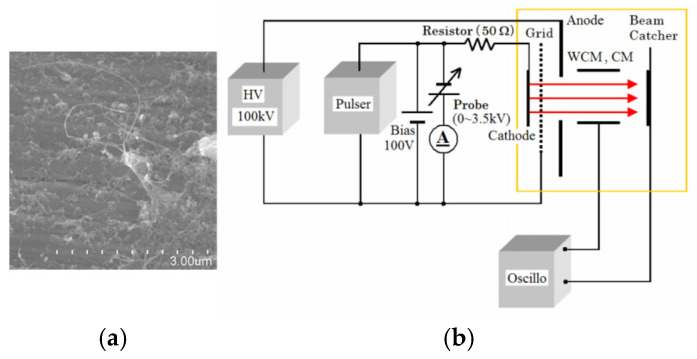
(**a**) SEM image of the CNT cathode; (**b**) The main components of the electron gun system [126].

Y. Hozumi et al. used a material consisting mostly of carbon-like spherical particles and a small amount of un-grown up CNTs as the cathode to develop an injector gun with a narrow energy distribution and high intensity electron beam [126], as shown in Figure 33. An anode current of 100 mA was obtained from the CNT cathode, corresponding to a current density of about 400 mA/cm^2^.

**Figure 34:**
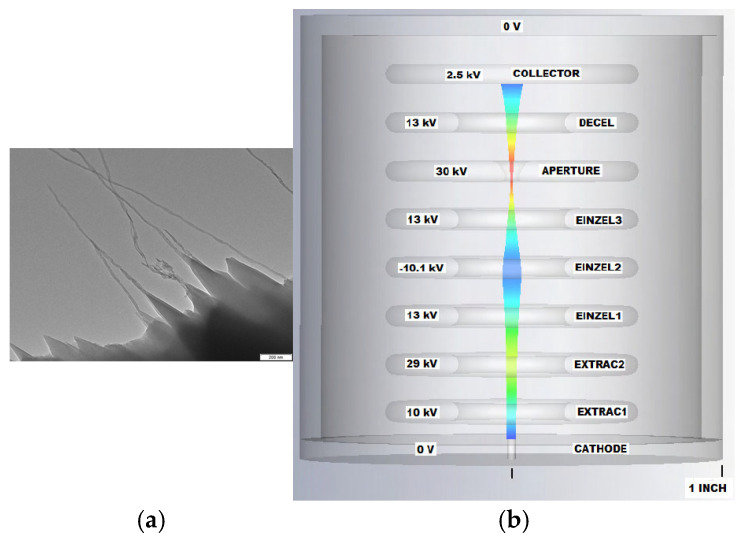
(**a**) TEM image of the Ar^+^-irradiated RVC. “200 nm” in lower right; (**b**) Simulation results of electrostatic focusing cold cathode electron gun [127].

**Figure 35:**
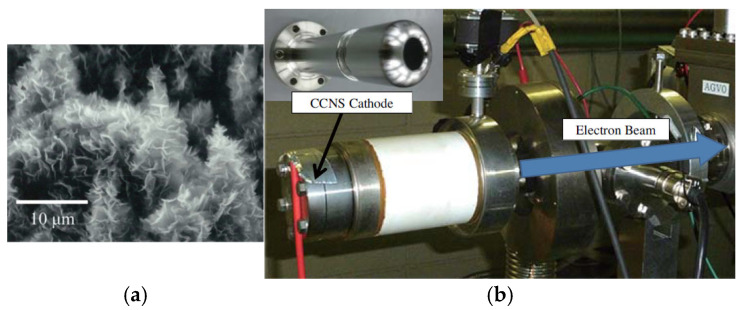
(**a**) SEM image of the coniferous-type carbon nanostructures; (**b**) Electron gun based on the CCNS with 16-mm-diameter cathode [130].

Brady C. Smith et al. used argon ion to irradiate reticulated vitreous carbon (RVC) and found a large number of graphene-rich nanostructures over the RVC surface, including single- and multi-walled CNTs [127], as shown in Figure 34. The cathode is fabricated using argon-ion-irradiated RVC. The cold cathode electron gun made with the RVC exhibits high current density [128,129]. The principal simulation results of the designed cylindrically-symmetric electron gun are shown in Figure 34b. Electrons are ejected from the cathode surface, then adjusted by the Einzel lens designed. The results indicate that the RVC cathode-based electron gun exhibits high current density, medium energy, and low energy diffusion. It was reported that coniferous-type carbon nanostructure (CCNS) can generate an electron current density of over 100 mA·cm^−2^ continuously for more than 1300 h [130,131]. Hidetoshi Kato et al. fabricated a novel electron gun that uses a CCNS cathode in a superconducting accelerator [130], as shown in Figure 35. CCNS is composed of a complex of CNT, nano-diamond, and graphene. The tip shape of CCNS is the same as that of CNT. When an electric field is exerted on CCNS, it concentrates at a tip, and electrons are ejected through field electron emission. An emission current density of 20 mA·cm^−2^ was observed. Stable electron emission was measured continuously for 1274 h, exhibiting only a slight increase. Electron guns based on CCNS cathode demonstrate promising potential for compact X-ray sources.

**Figure 36:**
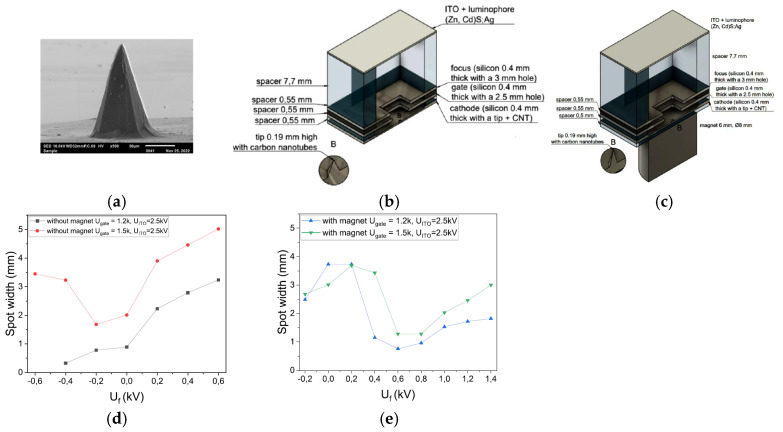
(**a**) Silicon tip covered with carbon nanotubes; (**b**) Measurement system for testing tip field emitters; (**c**) Measurement system for testing tip field emitters with magnetic focusing; (**d**) Dependence of the size of the electron beam spot in the function of the focus voltage; (**e**) The beam spot size depending on the focus voltage with a magnetic field [132].

Paweł Urbański et al. designed and fabricated a point FE electron source with its silicon tip covered with a layer of CNTs [132], as shown in Figure 36. The entire surface of the silicon tip was surrounded by deposited CNTs using a spotting method, forming a thin layer on top of it. The tip emitter can generate a current of several to tens of microamperes. The emitted beam can be focused into a small and homogeneous spot by an electrostatic lens and a magnetic field. The results of electron beam trajectories simulations under varying focus voltages revealed that the focal point can be adjusted by modifying the electrostatic field. They also analyzed and obtained optimal focusing at −200 V for the system without a magnet and 600 V for the system with a magnet. Furthermore, the transmission rate in each measurement is at least 95%. These results show the potential applications of the developed emitter in miniature X-ray sources and electron microscopy.

## 6. Microwave Electron Gun Based on CNT Cold Cathode

The cold cathode electron gun based on CNTs combined with microwave field modulation has demonstrated distinct advantages in microwave electronic device applications, such as improving input power transmission efficiency and developing fully integrated vacuum radiation sources.

**Figure 37:**
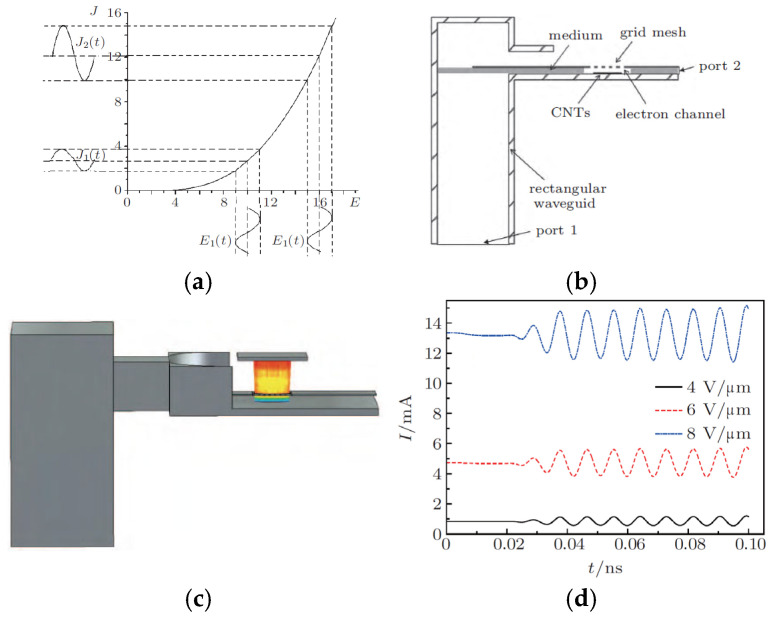
(**a**) FE beam current density as a function of electrostatic field under DC-shifted AC excitation; (**b**) Scheme of the cold cathode electron gun; (**c**) Scheme of the electron beam trajectories; (**d**) The FE beam currents modulated by HF fields of 114 GHz under different electrostatic fields [133].

Xingxing Li et al. designed an FE electron gun based on CNTs to study electron beam modulation, as shown in Figure 37 [133]. When a direct current (DC) electrostatic field E_0_ and a high-frequency (HF) field E_1_(t) act concurrently on the cold cathode, the FE beam current density consists of a dc component (J_0_) and a time-modulated alternating current (AC) component J_1_(t), as shown in Figure 37a. Different resulting J_1_(t) and J_2_(t) components and modulation depths were obtained for the increased electrostatic field E_0_ and the maintained source E_1_(t). When the electrostatic field is large, the AC beam current density component is also great. The magnitude of the dc source can amplify the AC output. The designed cold cathode electron gun is shown in Figure 37b. Parallel plates are employed as the cathode and anode of the electron gun. The grid mesh is positioned above the CNT cathode. Modulated electron beams are emitted through the grid holes in the anode. The waveguide in the cold cathode electron gun has a broad side of 2.54 mm and a narrow side of 1.27 mm. When an HF field is applied to the plates via the waveguide, port 1 (the waveguide port) serves as input, and port 2 (the microstrip) as output. Xuesong Yuan et al. also designed a specialized CNT cold cathode electron gun to investigate multi-beam modulation [134]. The electron gun comprises two waveguide ports: a high-frequency (HF) field is transmitted into the interelectrode gap through port 1 of the rectangular waveguide, and exits via port 2 on its right side. The TE_10_ mode of the HF field in the waveguide can be converted to a TEM mode within the parallel plate structure. There are five electron beams, each originating from a 5 × 5 CNT array cathode with an approximate size of 0.4 mm under the operating conditions. Then the converted HF field passes through the CNT cold cathodes and modulates the emission beams.

**Figure 38:**
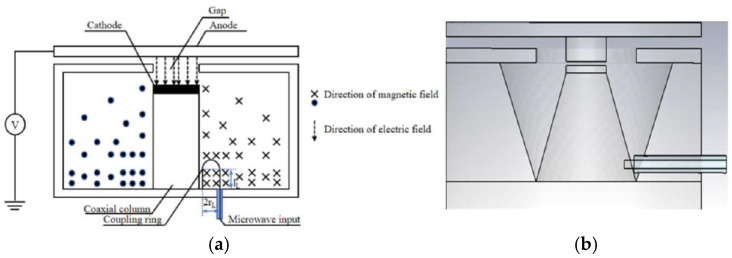
(**a**) Schematic of the reentrant resonator with TM_010_ mode field distribution; (**b**) The optimized cavity structure [136].

In addition, Jie Xie et al. also proposed a microstrip structure electron gun based on CNTs for an extended interaction frequency-locking oscillator [135]. A modulated electron beam can be obtained in the electron gun to lock the oscillation frequency. Given a 35 GHz input HF signal with powers of 5, 10, and 15 W (accompanied by a 625 V DC voltage), the electron beam currents are 126–170, 118–180, and 112–188 mA, and the modulation depths are 17.9%, 20.8%, and 25.3%, respectively. So a stronger input signal is applied to the electron gun, resulting in a higher HF field on the surface of the cold cathode and a greater modulation depth of the beam current. Jiaxin Li et al. proposed a CNT cold cathode microwave electron gun using a resonant cavity for high-frequency microwave vacuum electron devices [136], as shown in Figure 38. The proposed microwave cold cathode electron gun consists of a cold cathode, a cavity, and an anode collector. An optimized resonator cavity structure with a tapered gradient circular truncated cone cathode, a coupling coaxial line, and the anode plate with a cylinder extended down in the middle was also proposed, which could overcome the technical problem that the fabrication and assembly of a coupled ring with a size of around 1–2 mm, as shown in Figure 38b. Simulation results showed that at an input microwave power of 200 W, the maximum emission current is 1.14 A and the corresponding current density is 9.07 A/cm^2^, and the cathode surface electric field is up to 3.65 × 10^6^ V/m. The results indicated that the CNT cold cathode microwave electron gun can achieve ampere-scale current and ampere-scale current density per square centimeter under high-frequency microwave electric field modulation. The modulated electron beam based on a CNT cold cathode is of great significance to the development of miniaturized and integrated vacuum electron devices.

## 7. Conclusions

CNTs have become ideal cathode electrode materials due to their unique FE properties, including structural, electrical, and thermal properties, and have attracted significant attention in FE devices. CNT-based cold cathode electron guns with different morphology types of CNT cathode were introduced. These electron guns have been studied both theoretically and experimentally for a variety of applications. CNT cathodes have achieved substantial improvements in practical aspects such as macroscopic FE current, current density, and long-term stability. Significant progress has also been made in the research of electron guns with CNT cold cathode, laying the foundation and paving the way for their comprehensive applications.

Although CNT cold cathode electron guns have achieved good performances in recent years, there are still several issues that need to be further addressed, including the simultaneous high emission current and its density in reality, longer lifetime at high current density, stability under higher voltage, and high beam focusing quality. These issues may hinder the broader and deeper use of CNT-based practical devices. Precisely adjusting the electrical properties of CNT emitters and designing an efficient electron emission structure are the research challenges. Further developing the novel manufacturing technique of CNT emitters is a significant approach for enhancing the characteristics of CNT cathodes. Enhancing the adhesion of CNT emitters to substrates is a highly effective solution to get stable high current and high current density. Artificial intelligence, as a current work tool, can go some way in contributing to exploring and predicting the properties of CNT-based emitters. Systematic and in-depth optimization of the component structures of the CNT cold-cathode electron gun represents an essential pathway to devices with superior performance. For high-resolution X-ray sources and scanning electron microscopes, the CNT-based cold cathodes with high electron beam brightness and low energy spread remain crucial. Fabrication technologies of CNT-based cold cathode devices hold great potential in emerging applications such as high-frequency communications systems and space missions. The integration of CNT-based emitter platforms with micro-electro-mechanical systems is a promising approach for fabricating high-performance microwave vacuum electronic devices and micro-propulsion systems. In addition, the development of microwave devices with novel structures by harnessing the unique properties of CNT cathodes is also a valuable research area. With the joint efforts of scholars from all over the world, the characteristics and device applications of CNT cold cathode electron guns are expected to reach a new level soon.

## Data Availability

Data sharing not applicable.
